# Dutch-TIMELINESS: optimising choledocholithiasis treatment in the Netherlands – protocol of a national implementation project

**DOI:** 10.1136/bmjopen-2026-118615

**Published:** 2026-07-23

**Authors:** Anne J J The, Maud M T Merks, Robert C Verdonk, Lucas Goense, Max H G van Maasakkers, Peter J van der Schaar, Peter van Duijvendijk, Niels G Venneman, Mike S L Liem, Jan P Deroose, Willem J Thijs, Lea M Dijksman, Djamila Boerma, Teus J Weijs

**Affiliations:** 1 Department of Surgery, St Antonius Hospital, Nieuwegein, The Netherlands; 2 Department of Gastroenterology and Hepatology, St Antonius Hospital, Nieuwegein, The Netherlands; 3 Department of Surgery, Gelre Ziekenhuizen, Apeldoorn, The Netherlands; 4 Department of Gastroenterology & Hepatology, Medisch Spectrum Twente, Enschede, The Netherlands; 5 Department of Surgery, Medisch Spectrum Twente, Enschede, The Netherlands; 6 Department of Surgery, Martini Hospital, Groningen, The Netherlands; 7 Department of Gastroenterology & Hepatology, Martini Hospital, Groningen, The Netherlands; 8 Department of Research and Development, St Antonius Hospital, Nieuwegein, The Netherlands

**Keywords:** Endoscopy, GASTROENTEROLOGY, SURGERY, Clinical Protocols, Hepatobiliary disease

## Abstract

**Abstract:**

**Introduction:**

The Dutch guideline ‘Gallstone disease’ (2016) recommends performing a cholecystectomy within 72 hours following endoscopic retrograde cholangiopancreatography (ERCP) for common bile duct stones to prevent recurrent gallstone-related complications. Nevertheless, guideline adherence in the Netherlands remains low, and the time between ERCP and cholecystectomy often exceeds 72 hours. Our project aims to improve national guideline adherence to 85% or higher and thereby reduce the incidence of gallstone-related complications after ERCP.

**Methods and analysis:**

The Dutch-TIMELINESS is a multicentre implementation project using a Multiphase Optimisation Strategy. The project includes 61 Dutch hospitals. In phase 1, all patients undergoing ERCP for choledocholithiasis from 1 January to 31 March 2023 will be analysed to serve as a benchmark. In phase 2, each hospital develops and implements its own tailored protocol in line with the guideline, guided by the benchmark data. In phase 3, from 1 December 2025 to 31 March 2026, the new protocols will be prospectively evaluated with real-time monitoring and refined as necessary. Statistical methods are predefined in the protocol.

**Ethics and dissemination:**

The Medical Research Ethics Committees United reviewed the protocol and concluded it does not fall under the scope of the Medical Research Involving Human Subjects Act (WMO). Local institutional research departments and the board of directors reviewed and approved the protocol in all participating hospitals. Results will be published in international peer-reviewed scientific journals and presented at national conferences.

## Introduction and rationale

 Yearly, 30 000 patients are diagnosed with cholecystolithiasis in the Netherlands.^1 2^ These gallstones may migrate into the common bile duct (CBD) which is known as choledocholithiasis.^3^ The Dutch guidelines recommend treating symptomatic choledocholithiasis with papillotomy and stone extraction through endoscopic retrograde cholangiopancreatography (ERCP), followed by laparoscopic cholecystectomy within 72 hours.^4^ Despite this recommendation, adherence in clinical practice remains limited.

The evidence for routine cholecystectomy following ERCP for choledocholithiasis is extensive. In 2002, a randomised controlled trial (RCT) demonstrated that cholecystectomy following ERCP reduced the risk of recurrent gallstone complications from 47% to 2% compared with a wait-and-see approach.^5^ These findings have been replicated in other RCTs.^6–8^ Subsequent meta-analysis even showed a reduced risk of mortality for routine cholecystectomy compared with wait-and-see.^9^

Despite this strategy, approximately 20% of patients develop recurrent symptoms or complications while awaiting elective cholecystectomy. This can be prevented by early cholecystectomy, within 72 hours after ERCP.^10–14^ This timeframe avoids 10% redo ERCPs due to recurrent CBD stones in the waiting time and 14% emergency cholecystectomies due to cholecystitis.^14^ An early cholecystectomy following ERCP is also the most effective strategy in terms of costs, and therefore a significant argument in this time of increasing healthcare expenses.^15^

Performing CBD clearance and cholecystectomy as a single-session procedure instead of a two-step procedure is even more effective.^16–18^ This virtually eliminates the chance for recurrent biliary events and shortens hospital stay. However, the single-session procedure is logistically challenging in the Dutch healthcare system: the success rate of endoscopic removal of CBD stones is high, and subsequently, the experience with laparoscopic CBD exploration has ceased. For a single-session treatment, thus two specialists are required (gastroenterologist and surgeon), posing big logistical challenges. Therefore, in the Netherlands, the two-step procedure is standard.

The 72-hour timeframe for the two-step procedure has been recommended by the Dutch guidelines since 2016, but compliance remains low. Based on a short survey with 40 gastroenterologists from 40 hospitals in January 2024, it was estimated that only 10% of patients undergoing ERCP for choledocholithiasis receive a cholecystectomy within 72 hours. The underlying reasons for this low percentage may include the absence of standardised protocols, logistical challenges, unclear ownership of care, a lack of benchmark data and inadequate performance feedback.

To address the causes of poor guideline adherence and to improve compliance, the Dutch-TIMELINESS project was designed as a 2-year implementation project using a Multiphase Optimisation Strategy (MOST).^19^ The Dutch-TIMELINESS project aims to achieve that in 2 years’ time, 85% of patients receive a cholecystectomy within 72 hours following successful ERCP in the Netherlands. Hereby, we aim to reduce complications, re-interventions, emergency surgeries and associated healthcare costs.

## Methods

### Primary objective

To achieve adherence rates of over 85% for cholecystectomy within 72 hours following successful ERCP for choledocholithiasis in the Netherlands.

This goal of 85% is in line with the results of a similar project in the UK, set up to improve timely emergency cholecystectomy rates for cholecystitis. This project demonstrated that significant improvement is feasible within a 2-year timeframe. Through a quality improvement collaborative, the rate of performing emergency cholecystectomies within 7 days increased from 5% to 85% in 2 years.^20^

### Project design

This project is reported in accordance with the StaRI guidelines for implementation studies.^21^ The Dutch-TIMELINESS is a national before-after implementation project comprising 61 hospitals performing ERCP in the Netherlands. This implementation project is designed using the Updated Consolidated Framework of Implementation Research (CFIR) and the strategies mentioned in the strategy tool from The Netherlands Organisation for Health Research and Development. These strategies are based on the CFIR-ERIC matching tool.^22 23^ The CFIR is a listing of constructs with potential influencing factors for implementation, developed by Damschroder *et al.* in 2009.^24^ By using the CFIR, barriers and facilitators of the implementation process will be identified. During the conduction of the Dutch-TIMELINESS, the implementation process will be evaluated and readjusted at predefined time points, if needed.

### Description

The MOST is adopted as implementation strategy.^19^ This strategy consists of three subsequent phases: preparation, optimisation and evaluation. The Dutch-TIMELINESS project will be organised in three phases across 24 months, accordingly.

#### Phase 1: baseline measurement, determining barriers and providing performance feedback

The objective of the first phase is to provide centre-specific performance feedback to all participating hospitals, based on their local baseline measurement. Furthermore, this phase enables us to identify best practices, additional local barriers and local opportunities. The Dutch-TIMELINESS project will retrospectively analyse all patients that underwent ERCP for choledocholithiasis in the period between 1 January 2023 and 31 March 2023. Data regarding patient, treatment and outcome characteristics will be extracted from the patient’s electronic health record. Frailty will be measured using the validated modified Frailty Index-5 (mFI-5).^25^ We decided on using this frailty index as it was proven a strong predictor of mortality and postoperative complications in geriatric patients undergoing cholecystectomy.^26^

The data collection in phase 1 will be executed conform the Snapshot method.^27^ The Dutch Snapshot Research Group (DSRG) is a Dutch surgical network that facilitates research collaboration in the Netherlands, initiated in 2014. The Snapshot research method is collaborative research with the aim of easily collecting data from multiple hospitals, with help from residents in each centre. For that reason, the approval of the DSRG was obtained prior to the start of the Dutch-TIMELINESS. For this first phase, data has been collected since February 2025. The timing of data completion differed across participating centres, with some centres completing their registrations earlier than others.

#### Phase 2: engaging local stakeholders and establishing tailored guidelines

The objective of this second phase is to establish a tailored protocol for the treatment of choledocholithiasis in each participating hospital. First, the participating hospitals will receive performance feedback based on their baseline measurement. This feedback will contain the local percentage of guideline adherence compared with the average results and best practices. The hospitals that perform best will be asked to share their protocols and policies around choledocholithiasis. The summary provided to each hospital will also include their own essential data needed for logistics, such as the mean number of patients per week, the number of referred patients, the success rate of the ERCP and the time between diagnosis and ERCP. The latter may be key, as proper planning and teamwork between gastroenterologist and surgeon from diagnosis onwards will buy valuable time. Second, guided by the summarised data, local barriers and determinants of success will be found. Each hospital will gather their local stakeholders. These stakeholders will at least consist of the gastrointestinal surgeons, gastroenterologists and anaesthesiologists. Together, they will establish a local, tailored guideline. All parties involved will be informed by the local investigators and will be actively included in the process.

#### Phase 3: evaluation and adjustments

The objective of this third phase is to evaluate the adherence to the newly established guideline. The aim is to increase the percentage of patients undergoing cholecystectomy within 72 hours after ERCP to at least 85%. During this phase, local investigators will prospectively include patients undergoing ERCP in the period from 1 December 2025 to 31 March 2026. Data regarding patient, treatment and outcome characteristics will be extracted from the patient’s health record. Every 4 weeks, the use of the newly established local guideline will be evaluated and, if necessary, adjusted by a designated gastroenterologist and surgeon.

**Figure 1:**
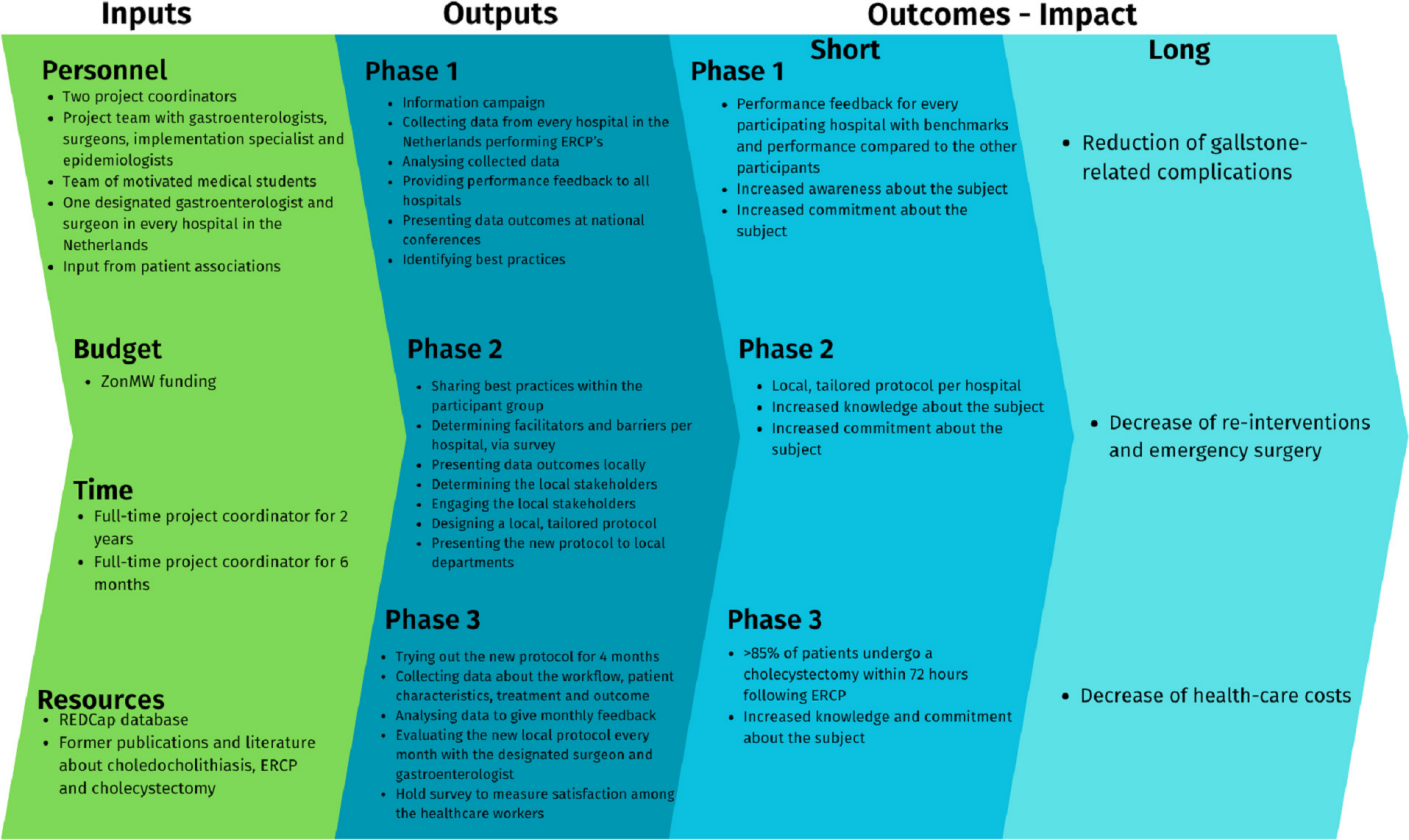
Logic model of the TIMELINESS project. The model displays the inputs, outputs and short- and long-term outcomes across three phases. ERCP, endoscopic retrograde cholangiopancreatography; ZonMW: the Dutch organisation for knowledge and innovation in health, healthcare and well-being.

**Figure 2:**
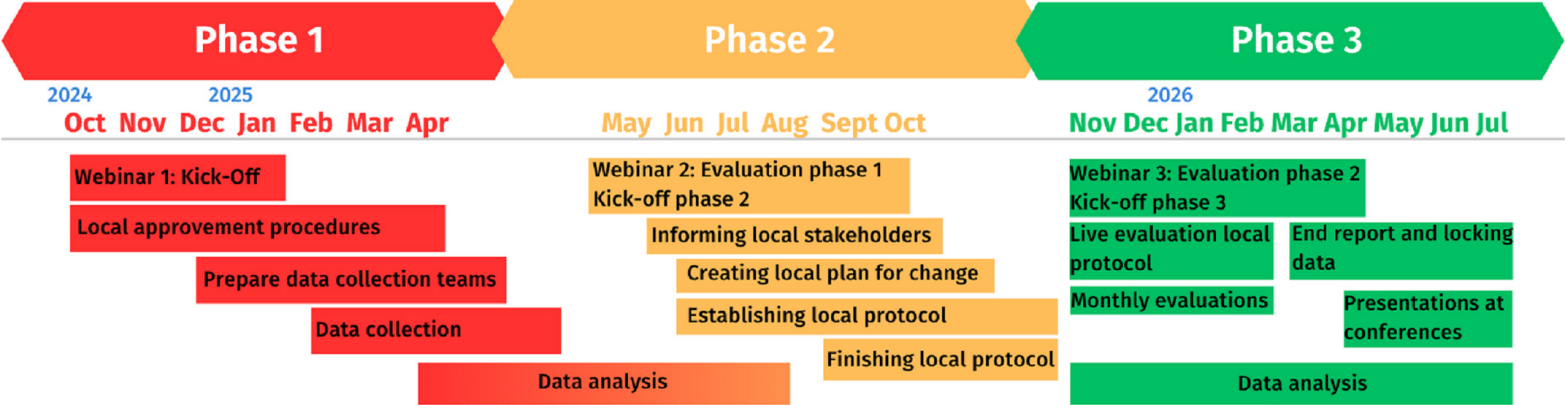
Timeline with events. The timeline illustrates the three phases of the TIMELINESS project and lists key activities for each phase.

To visualise a detailed description of the implementation process, we developed a logic model^28^ (figure 1) and a timeline with events (figure 2).

#### Information campaign

An information campaign will be held throughout the duration of the project, as briefly outlined in the logic model and the timeline (figures 1 and 2). A series of webinars will be organised to inform and engage participants during phases 1, 2 and 3. Information will be disseminated through presentations at local and national conferences. The project’s aim and protocol will be published in a variety of professional magazines and newsletters targeting the key stakeholders, including surgeons, gastroenterologists, anaesthesiologists, endoscopy nurses and operating room nurses. Additionally, the treatment strategy (cholecystectomy within 72 hours following ERCP) will be incorporated in the biannual course on biliary disease for surgical residents. Finally, the treatment strategy will be highlighted in information leaflets for patients with gallstone disease, provided by the Dutch Digestive Health Fund. During the entire process, updates and information will be spread via social media platform LinkedIn and via email to keep the whole network engaged.

### Targeted sites, population and eligibility criteria

In the Netherlands, 62 hospitals perform ERCPs. This project comprises a total of 61 hospitals, including 60 hospitals that perform ERCPs. One hospital that does perform ERCP is not involved, as they are not performing ERCP for choledocholithiasis. One regional hospital that does not perform ERCPs but has a tight collaboration with a hospital in Belgium that performs ERCPs for their patients has been included. Since they had a strong incentive to optimise their care for these patients, they were included in the project. This makes a total of 61 hospitals included in the project. Six of the 61 hospitals are academic hospitals and part of the Netherlands Federation of University Medical Centres. 28 of the remaining 55 hospitals are part of the Association of Tertiary Medical Teaching Hospitals. 26 hospitals are regional hospitals. Finally, one of the participating hospitals is a specialised cancer institute that also performs ERCP for choledocholithiasis and was therefore considered eligible for inclusion.

For the first phase, all patients aged 18 years or older who underwent ERCP for choledocholithiasis in the period between 1 January 2023 and 31 March 2023, with a gallbladder in situ, will be included. This period was selected because it was thought to accurately represent the best everyday practices. COVID-19 was no longer active, and there were no significant holidays that might have interrupted operation schedules. Moreover, the Dutch-TIMELINESS could not have influenced results, since the project was not yet pitched or designed. Patients included in this period will be followed for 2 years after the baseline ERCP. This extended follow-up will be used for secondary and descriptive analysis of recurrent biliary problems. The primary before-after comparison will focus on guideline adherence within 72 hours after successful ERCP.

In phase 2, no patient data will be collected.

In phase 3, all patients 18 years or older diagnosed with choledocholithiasis, with a gallbladder in situ, who will undergo ERCP in the period between 1 December 2025 and 31 March 2026, will be included. This period was chosen to match the calendar quarter used in phase 1. December will be used as a wash-in period to separate the start-up effects from the true effect of the implementation. Data from this period will be excluded from the analysis to minimise bias from partial adoption of the local protocol. The follow-up period for phase 3 will be 8 weeks from the date of ERCP, to ensure more complete data on elective cholecystectomies and postoperative complications. A prolonged follow-up is not required as the primary aim of this phase is to evaluate the implementation process rather than long-term outcomes.

For the primary analysis, the population will be defined identically in both phases. All eligible patients with a gallbladder in situ who underwent a first successful ERCP for choledocholithiasis within the predefined inclusion window. Patients who are not candidates for cholecystectomy due to surgical contraindications will be excluded from the analysis. These surgical contraindications include absolute contraindications for general anaesthesia (such as haemodynamic instability or extensive comorbidities) as determined by the attending anaesthesiologist, liver cirrhosis, loco-regional malignancy, gallbladder fistula and extensive or complicated previous abdominal surgery. These patients will be recorded in the database but excluded from outcome analysis. Patients who decline cholecystectomy or are advised against cholecystectomy for non-surgical reasons will remain included. Patients who are transferred will be registered in the hospital where the successful ERCP was performed. Patients who are transferred solely for cholecystectomy will also be registered under the ERCP hospital. This approach ensures that the primary endpoint is evaluated in comparable populations in both periods.

### Sample size

Dutch-TIMELINESS is a national implementation project rather than a conventional trial designed primarily to test a treatment-effect hypothesis. Because the expected difference is large, a conventional superiority calculation comparing the increase from approximately 10% to 85% would require only seven patients (two-sided alpha of 0.05 and a power of 0.80). If this minimum number were applied to each of the 61 participating hospitals, this would correspond to 427 patients per period. However, this calculation is mainly illustrative, as the primary aim is the national estimation of adherence around the predefined implementation target.

In 2023, a total of 9389 ERCP procedures for choledocholithiasis were recorded in the Netherlands.^29^ These were performed on 8545 unique patients, indicating that some underwent multiple ERCPs. With 61 hospitals participating, 3 months of data collection is expected to yield 35 patients per hospital (2135 patients in total). Within a total of two periods, assuming an anticipated 10% non-inclusion, the project is expected to include approximately 3843 patients. With an average cluster size of 35 patients, and an assumed intracluster correlation coefficient of 0.10, the design effect is 4.40. At an expected post implementation adherence of 85%, this allows estimation of national adherence with a 95% CI of approximately 85%±3.4 percentage points. The planned sample provides sufficient precision to estimate adherence around the predefined implementation target, supports hospital-level benchmarking and allows for adjusted analyses accounting for clustering and case mix.

### Patient and public involvement statement

The Nederlandse Leverpatiënten Vereniging (Dutch Liver Patient Association) was involved from study design onwards. They were asked to ensure patient perspective and contribute to the development of centralised patient information. They shared their thoughts during the project concept presentation and gave feedback on the trial protocol. Their input confirmed that the Dutch-TIMELINESS project is relevant for patients.

## Outcome measures and predefined statistical analysis plan

### General principles

The gathered data during phase 1 and phase 3 will be stored and managed using REDCap electronic data capture tools.^30 31^ Data analyses will be performed using the latest version of RStudio.^32^ Data will be checked for inconsistencies and missing values, which will be corrected or retrieved when possible. If necessary, missing data will be imputed using multiple imputation. Descriptive categorical data will be presented as counts and percentages. Categorical variables will be analysed using the χ^2^ test or Fisher’s exact test. Continuous data will be presented with means, SD and percentiles. Where appropriate, comparisons will be made using Mann-Whitney U tests, Kruskal-Wallis tests, t-tests or analysis of variance.

### Primary objectives’ statistical plan

To determine whether this implementation project has improved guideline adherence, we will perform a before-and-after analysis as previously described in the methods section. The primary outcome measure is adherence to the Dutch National Guideline ‘Gallstone disease’,^4^ defined as the proportion of patients undergoing cholecystectomy within 72 hours after successful ERCP. These percentages will be reported per hospital (anonymously) and as national mean. The results will be depicted using a slope chart showing the change in guideline adherence per hospital between the two time points we will compare: January to March 2023 versus January to March 2026. The proportion of hospitals reaching the predefined 85% adherence target will also be reported.

Due to the non-randomised nature of this project, we expect differences in baseline characteristics between hospitals and time periods. The primary statistical analysis will use a mixed-effects logistic regression model fitted at patient level. Period defined as year 2023 versus 2026 will be included as the fixed effect of primary interest. To adjust for case-mix, prespecified variables will be included as fixed effects. These include: age, body mass index, American Society of Anaesthesiologists classification, history of biliary disease, history of abdominal surgery, use of anticoagulation, use of immunosuppressants, mFI-5 score and dementia. A random intercept per hospital will account for clustering. To improve clinical interpretability, marginal model-based adjusted adherence rates for each period and the adjusted absolute difference in adherence between periods will also be derived from the mixed-effects model and reported with 95% CIs.

Missing data will be handled using multiple imputation under the assumption of missing-at-random. The imputation model will include the outcome, period, hospital, all variables included in the analysis model. The number of imputations will be at least 20. Estimates will be pooled according to Rubin’s rules. Prespecified sensitivity analysis will include: a complete case analysis, an unadjusted mixed-effects model including period as the only fixed effect, a hospital-level paired analysis in which the change in adherence is calculated for each hospital and summarised nationally.

During the process of project design, several questions of scientific interest were encountered that will be addressed, using the data generated by the Dutch-TIMELINESS project. These predefined secondary objectives, along with a brief plan for their analysis, are presented in online supplemental file 1. These secondary objectives include:

- To determine the effect of the time between diagnosis of choledocholithiasis and ERCP on morbidity, the success rate of the first ERCP, complications during ERCP and post-ERCP.
- To determine the effect of the time between ERCP and cholecystectomy on morbidity and surgical complications.
- To determine whether frailty modifies the association between cholecystectomy within or after 72 hours and morbidity in frail patients.
- To determine whether gallstones on preoperative imaging are associated with recurrent biliary events before cholecystectomy and with the presence of intraoperative gallbladder stones.
- To compare national hospital-level healthcare costs before and after the Dutch-TIMELINESS project.
- To evaluate the adoption, implementation and maintenance of the Dutch guidelines following the Dutch-TIMELINESS project.

**Strengths and limitations of this study**

- National coverage: participation of all centres in the Netherlands performing endoscopic retrograde cholangiopancreatography for choledocholithiasis.
- The phased implementation strategy benchmarks current practice, customises protocols and evaluates results to enable local changes while following national guidelines.
- Before-after design: system changes over time might influence adherence independent of the intervention.
- Selection bias: non-participation in the prospective phase might lead to potential selection.
- Information bias: in the retrospective phase, loss to follow-up might occur.

## Ethics and dissemination

The Medical Research Ethics Committees United (MEC-U) Reference number: W24.123, reviewed the protocol and concluded it does not fall under the scope of the Medical Research Involving Human Subjects Act (WMO). In phase 1, data are collected anonymously and, therefore, in accordance with the Dutch Agreement on Medical Treatment Act, a consent procedure could be deferred.

In phase 3, patient data that are generated during routine care will be collected prospectively. Most patients have a short hospital stay and typically have only one follow-up contact (a telephone call or an outpatient visit). Obtaining written informed consent for registration and data collection from all these eligible patients within the required time frame presents logistical challenges and may introduce participation bias.^33 34^ Furthermore, based on these challenges and the large sample size, the legal department of St. Antonius Hospital advised that written informed consent for phase 3 could be waived if an opt-out procedure was implemented in accordance with the Dutch Agreement on Medical Treatment Act. Therefore, the opt-out procedure was implemented into the protocol.

At every participating hospital, the local institutional research department and the board of directors also reviewed and approved the protocol. In accordance with local policy and applicable regulations, hospitals applied the waived informed consent for phase 1 and one of the following procedures for phase 3: (1) written informed consent (n=3), (2) verbal informed consent (documented in the medical record) (n=8) or (3) an opt-out (objection) procedure for use of patient data (n=50). Under the opt-out procedure, eligible patients are informed about the project and given the opportunity to object to data collection; data from patients who object is excluded.

## Dissemination

Each participating hospital will receive their own performance data set against the nationwide average and compared with the best practices. Results of the Dutch-TIMELINESS project will be submitted for publication in international peer-reviewed scientific journals and will be presented at national conferences.

### General

This study is not prospectively registered in a clinical trial registry. The complete protocol is presented in this manuscript in accordance with the StaRI reporting guidelines to ensure transparency and reproducibility.

## Supplementary material

10.1136/bmjopen-2026-118615 online supplemental file 1
