# Supplementary material for: Dutch-TIMELINESS: optimising choledocholithiasis treatment in the Netherlands – protocol of a national implementation project
Source: BMJ Open. 2026 Jul 23;16(7):e118615. doi: 10.1136/bmjopen-2026-118615 (PMC13404623; doi:10.1136/bmjopen-2026-118615)
Supplement: online supplemental file 1 [file bmjopen-16-7-s001.pdf]

**SUPPLEMENTARY APPENDICES**

## Title

# The Dutch-TIMELINESS: Optimising choledocholithiasis treatment in the Netherlands - protocol of a national implementation project

A.J.J. The[1], M.M.T. Merks[1], R.C. Verdonk[2], L. Goense[1], M.H.G. van Maasakkers[1], P.J. van der Schaar[2], P. van Duijvendijk[3], N.G. Venneman[4], M.S.L. Liem[5], J. P. Deroose[6], W.J. Thijs[7], L.M. Dijksman[8], D. Boerma[1], T.J. Weijs[1]

1. Department of Surgery, St. Antonius Hospital, Nieuwegein, The Netherlands
2. Department of Gastroenterology, St. Antonius Hospital, Nieuwegein, The Netherlands
3. Department of Surgery, Gelre Ziekenhuizen, Apeldoorn, The Netherlands
4. Department of Gastroenterology, Medisch Spectrum Twente, Enschede, The Netherlands
5. Department of Surgery, Medisch Spectrum Twente, Enschede, The Netherlands
6. Department of Surgery, Martini Hospital, Groningen, The Netherlands
7. Department of Gastroenterology, Martini Hospital, Groningen, The Netherlands
8. Department of Research and Development, St Antonius Hospital, Nieuwegein, The Netherlands

**Corresponding author**

A.J.J. The; Department of Surgery, St. Antonius Hospital, PO Box 2500, 3430EM, Nieuwegein, The Netherlands, email: a.the@antoniushospital.nl

**ORCHID-ID:** 0009-0008-5231-2256

## Index

**Secondary objectives** page 2.

**Table 1: Definitions of clinical, economic and implementation outcomes** page 3.

**Secondary objectives' statistical plan** page 5.

**Figure 3: RE-AIM matrix** page 7.

**References** page 8.

**Secondary objectives**

1. To determine the effect of the time between diagnosis of choledocholithiasis and ERCP on:
    - Morbidity: a composite outcome including all (recurrent) biliary problems, unplanned hospital (re)admissions, emergency presentations due to gallstone disease during this interval, and complications of cholecystectomy. Each component of this endpoint will also be analysed separately.
    - Success rate of the first ERCP: a successful ERCP will be defined as a common bile duct free of stones and sludge after ERCP.
    - Complications during ERCP or post-ERCP, defined as complications within 30 days following ERCP, conform to the AGREE grading system.

2. To determine the effect of the time between ERCP and cholecystectomy in the Netherlands on:
    - Morbidity, defined as the composite outcome comprising all (recurrent) biliary problems and unplanned hospital (re)admissions, or emergency presentations due to gallstone disease during this interval.
    - Surgical complications, defined as intra-operative complications and post-operative complications, conform to the Clavien-Dindo grading system.

3. To determine whether frailty modifies the association between cholecystectomy within or after 72 hours and morbidity, in frail patients.

4. To determine whether gallstones on pre-operative imaging are associated with recurrent biliary events before cholecystectomy and with the presence of intraoperative gallbladder stones.

5. To compare national hospital-level health-care costs before and after the Dutch-TIMELINESS project.

6. To evaluate the adoption, implementation and maintenance of the Dutch guidelines following the Dutch-TIMELINESS project.

For a clear overview of defined outcomes see Table 1

| CATEGORY | OUTCOME | DEFINITION | TIMEPOINT | DATA SOURCE | UNIT OF ANALYSIS |
|---|---|---|---|---|---|
| **CLINICAL/IMPLEMENTATION** | Adherence to the Dutch National Guideline 'Gallstone disease' | Proportion of patients undergoing cholecystectomy within 72 hours after successful ERCP | Within 72 hours after successful ERCP | Medical records from phase 3 | Patient |
| **CLINICAL** | Morbidity (diagnosis to ERCP) | A composite outcome comprising all (recurrent) biliary problems and unplanned hospital (re)admissions, or emergency presentations due to gallstone disease during this interval | From diagnosis to ERCP | Medical records | Patient |
| **CLINICAL** | Success rate of first ERCP | A common bile duct free of stones and sludge after ERCP | Index ERCP | Endoscopy reports | Procedure |
| **CLINICAL** | ERCP complications | Complications within 30 days following ERCP, conform to the AGREE grading system | Within 30 days after ERCP | Medical records | Patient |
| **CLINICAL** | Morbidity (ERCP to cholecystectomy) | A composite outcome including all (recurrent) biliary problems, unplanned hospital (re)admissions, emergency presentations due to gallstone disease during this interval, and complications of cholecystectomy | From ERCP to cholecystectomy | Medical records | Patient |
| **CLINICAL** | Surgical complications | Intra-operative complications and post-operative complications, conform to the Clavien-Dindo grading system | Until Follow-up: within 8 weeks from ERCP | Medical records and surgical records | Patient |
| **CLINICAL** | Morbidity (Frail patients conform mFI-5) | Overall morbidity as defined above, compared between frail patients undergoing cholecystectomy within 72hours vs. after 72hours | ERCP to cholecystectomy | Medical records and surgical records | Patient |
| **CLINICAL** | Recurrent biliary events i.r.t. imaging findings | Time to first recurrent biliary event from ERCP, stratified by gallstone presence on imaging | ERCP to first event or censoring | Medical records | Patient |

| | | | | | |
|---|---|---|---|---|---|
| **ECONOMIC** | Costs | Direct medical expenses borne by hospital (length of stay, duration of surgery, re-interventions, outpatient visits, emergency department visits) | During index admission and follow-up | Medical records | Patient |
| **IMPLEMENTATION** | Implementation | Implementation success is defined by the predefined RE-AIM process indicators, see Figure 3 | Post-implementation period | RE-AIM data collection, see Figure 3 | Cohort |

*Table 1: Definitions of clinical, economic and implementation outcomes*

*ERCP: Endoscopic Retrograde CholangioPancreatography, i.r.t: In relation to, RE-AIM: Reach, Effectiveness, Adoption, Implementation, Maintenance.*

**Secondary objectives' statistical plan**

1. The time interval between diagnosis and ERCP will be presented as median (Q1, Q3) in days and, additionally, in hours. Associations with the success of the first ERCP (success is defined as the common bile duct free of stones and sludge), procedural or post-ERCP complications, and overall morbidity will be assessed using separate multivariable logistic regressions, with time modelled continuously and adjusted for pre-specified confounders. The list of confounding variables is provided at the end of Secondary Objective 4.

2. The interval between ERCP and cholecystectomy will be presented as median (Q1, Q3) in days and, additionally, in hours. Associations with overall morbidity and post-operative complications will be analysed using multivariable logistic regression, with time categorised as ≤72 hours versus >72 hours and adjusted for pre-specified confounders (provided at the end of objective 4). Sensitivity analyses will be performed, modelling time continuously. Additionally, time-to-event analyses will be performed using Cox proportional hazards models to analyse time to morbidity, with censoring at cholecystectomy.

3. A comparison will be made between frail patients undergoing cholecystectomy ≤72 hours and those undergoing cholecystectomy >72 hours, regarding overall morbidity. To improve comparability, propensity score matching will be applied using 1:1 nearest-neighbour matching, as an imbalance is expected due to the retrospective design of phase 1. Matching will be based on age, BMI, ASA classification, mFI-5 index, anticoagulation use, comorbidities, and functional status.

4. The number of patients with no evidence of gallbladder stones on pre-operative imaging and the proportion of recurrent biliary events within this group will be reported using descriptive statistics. Furthermore, the presence of gallbladder stones at the time of cholecystectomy will be summarised as observed proportion with corresponding percentage. The association between recurrent biliary events and the presence/absence of gallstones on imaging will be analysed using a Cox proportional hazards model, with follow-up from date of ERCP to first event. Patients will be censored at cholecystectomy, death, loss to follow-up or end of the project period.

Possible confounding variables that will be addressed in the regression analyses include: age, body mass index, American Society of Anaesthesiologists classification, history of biliary disease, diabetes, cardiovascular disease, pulmonary disease, history of abdominal surgery, use of anticoagulation, use of immunosuppressants, liver cirrhosis, dementia, and functional status.

5. After data collection in phase three of this project, a cost-effectiveness analysis will be performed. We will compare phases 1 and 3 from the hospital perspective. Only the direct medical expenses borne by the hospital will be evaluated, comprising length of stay, duration of surgery, re-interventions, outpatient visits, and emergency department visits. This

information will be extracted from the electronic health records from the two data collection phases. The expenses will be valued using hospital unit costs (EUR, 2025) assuming all care was delivered in the same hospital. The primary outcome is costs per acute readmission prevented. Additionally, incremental cost-effectiveness ratios (ICERs) will be calculated. To check for the accuracy of the results, sensitivity analyses will be performed. Results will be reported in accordance with the CHEERS guidelines.

6. Implementation outcomes will be evaluated using the Reach, Effectiveness, Adoption, Implementation, Maintenance (RE-AIM) framework (Figure 3) and predefined process indicators.[1]

Successful implementation will be defined by these predefined RE-AIM process indicators. Reach and Adoption will be measured using the same indicators, defined as the number of participating hospitals in phase one and phase three, the number of participants during webinars, and the number of views of webinars and LinkedIn posts.

Effectiveness will be defined by our primary objective: the proportion of patients undergoing cholecystectomy following ERCP within 72 hours.

Implementation will be assessed at hospital level based on development of a local protocol consistent with the national guideline, actual use of this protocol, engagement of key stakeholders, and fidelity to the original implementation plan. Fidelity will be assessed by determining whether planned activities (disseminating information in the own hospital, monthly evaluations conducted) are delivered as intended and whether a local protocol is designed. This will be checked by correspondence with the participating hospitals. The impact of real-time feedback during phase 3 will be assessed by comparing the adherence to the protocol at the sequential time points (after month 1- 4) and by documenting protocol changes.

Adaptations to the implementation strategy will be documented throughout the project phases through ongoing communication with the participating hospitals, including the monthly evaluation updates, email correspondence, webinar discussions and feedback.

Maintenance will be evaluated through surveys assessing the use of the protocol and intention of maintenance after project completion. Finally, maintenance will be facilitated by incorporating the timely cholecystectomy as a quality criterion in the Dutch gallbladder and ERCP registries.

For a clear overview of indicators and data sources, see Figure 3 for the RE-AIM matrix.

| RE-AIM stadium | Indicator | Data source | | | | | | | |
|---|---|---|---|---|---|---|---|---|---|
| | | Logdata | Redcap database phase 1 | Redcap database phase 3 | Protocol per hospital | Presentation per hospital | Observation use of whatsapp groups | Survey best and least performing hospital | Survey evaluation phase |
| Reach/Adoption | Number of participating hospitals phase 1 | ● | ● | | | | | | |
| | Participants webinars | ● | | | | | | | |
| | Number of participating hospitals phase 3 | ● | | ● | | | | | |
| Implementation | Determining facilitators and barriers | | ● | | | | | ● | |
| | Communication between hospitals | | | | | | ● | | |
| | Involvement of local stakeholders | | | | ● | ● | | | |
| | Use of new local protocol | | | ● | ● | | | | |
| | Number of monthly evaluations | ● | | | | | | | |
| | Fidelity to the implementation | | | | ● | ● | ● | | |
| Effectiveness | Median/mean time waiting period | | ● | ● | | | | | |
| | Number of gallstone-related events during interval | | ● | ● | | | | | |
| | Percentage of guideline adherence | | ● | ● | | | | ● | |
| | Satisfaction personnel | | | | | | | ● | ● |
| | Fidelity to the protocol | | | ● | | | | | ● |
| Maintenance | Intention for maintenance | | | | | | | | ● |
| | Use of protocol after evaluation phase ended | | | | ● | | | | ● |

*Figure 3 RE-AIM matrix. Each dot represents the data source used to measure the corresponding indicator. RE-AIM, Reach, Effectiveness, Adoption, Implementation, Maintenance.*

## References

1. Glasgow RE, Vogt TM, Boles SM, Glasgow E. Evaluating the Public Health Impact of Health Promotion Interventions: The RE-AIM Framework [Internet]. Vol. 89. 1999. Available from: www.ori.
