# Supplementary material for: Dutch-TIMELINESS: optimising choledocholithiasis treatment in the Netherlands – protocol of a national implementation project
Source: BMJ Open. 2026 Jul 23;16(7):e118615. doi: 10.1136/bmjopen-2026-118615 (PMC13404623; doi:10.1136/bmjopen-2026-118615)
Supplement: Supplementary file 2 [file bmjopen-2026-118615.reviewer_comments.pdf]

PEER REVIEW HISTORY

BMJ Open publishes all reviews undertaken for accepted manuscripts. Reviewers are asked to complete a checklist review form and are provided with free text boxes to elaborate on their assessment. These free text comments are reproduced below.

ARTICLE DETAILS

Title (Provisional)

The Dutch-TIMELINESS: Optimising choledocholithiasis treatment in the Netherlands - protocol of a national implementation project

Authors

The, Anne JJ; Merks, Maud MT; Verdonk, Robert C; Goense, Lucas; van Maasakkers, Max HG; van der Schaar, Peter J; van Duijvendijk, Peter; Venneman, Niels G; Liem, Mike SL; Deroose, Jan P; Thijs, Willem J; Dijksman, Lea M.; Boerma, Djamila; Weijs, Teus J

VERSION 1 - REVIEW

**Reviewer**	**1**
**Name**	**Han, Jimin**
**Affiliation**	**Daegu Catholic University School of Medicine, Department of Internal Medicine**
**Date**	**23-Mar-2026**
**COI**	**None**

This is a timely and potentially valuable protocol for a national implementation project addressing evidence–practice gap in the management of choledocholithiasis. The rationale is clinically strong, the multicenter national scope is a major strength, and the manuscript is clearly written overall. The protocol also appropriately frames itself as an implementation project using CFIR, MOST, and a before–after design across participating hospitals. Because this is a protocol, result-oriented StaRI items are understandably not yet applicable. The following are suggestions for both major and minor revisions:

Major revisions

1. It would be great to clarify and harmonize the analytic population across study phases.

This is the most important issue. In phase 1, the manuscript states that all patients undergoing ERCP for choledocholithiasis during the baseline period will be analyzed, whereas in phase 3 the evaluable population appears more closely linked to patients eligible for the intended care pathway and the primary endpoint is defined as cholecystectomy

within 72 hours after successful ERCP. As written, it is not fully clear whether the denominator is identical across phases, particularly with respect to failed ERCP, patients with contraindications to surgery, and transferred patients. This raises concern about comparability in the before–after analysis. The manuscript would be strengthened by defining one consistent analytic population and applying it identically in both phases.

2. Providing a more rigorous sample size justification and a more fully specified statistical analysis plan would be helpful for the potential readers and researchers.

The manuscript reports expected hospital participation and projected patient numbers and states that mixed-effects modeling will be used to account for clustering and case-mix. However, the sample size justification remains pragmatic rather than analytical, and the statistical section would benefit from more detail. In particular, the authors should specify the model structure more explicitly, including fixed and random effects, handling of hospital-level clustering, prespecified confounders, missing-data strategy, and planned sensitivity analyses. Since the study is non-randomized and multicenter, this level of detail is important for reader confidence.

3. Please prespecify fidelity, adaptation, maintenance, and registration information more clearly.

The supplement notes that adoption, implementation, and maintenance will be evaluated, and the main text states that local guidelines will be reviewed every four weeks and adjusted if necessary. However, the protocol does not yet clearly define how fidelity to the implementation strategy will be measured, how adaptations will be documented, or which process metrics will indicate successful implementation. In addition, the StaRI checklist includes study registration under general reporting items, but a registration platform/number or a clear explanation for non-registration is not readily visible in the manuscript. These points should be addressed explicitly.

Minor revisions

1. Adding an outcome definition table would improve readability.

Several outcomes are defined across the main manuscript and supplement, including successful ERCP, morbidity, surgical complications, cost outcomes, and implementation outcomes. A single consolidated table summarizing each outcome, its definition, timepoint, data source, and unit of analysis would improve readability and reduce ambiguity.

2. Please specify the contribution of patient and public involvement.

The manuscript states that the Dutch Liver Patient Association provided input on patient-related outcomes. It would be helpful to specify what, concretely, changed as a result of that input.

3. There are rooms for improvement regarding terminology and consistent use of words.

Examples include consistent use of "StaRI" capitalization, clearer wording around hospital numbers and ERCP capability, and minor language edits throughout. These are not major concerns, but they would improve readability of the protocol.

I look forward to implementation of the current protocol and thus future improvement in the managment of choledocholithiasis.

VERSION 1 - AUTHOR RESPONSE

Reviewer's comments to the authors

Reviewer: 1

Dr. Jimin Han, Daegu Catholic University School of Medicine

Comments to the Author:

This is a timely and potentially valuable protocol for a national implementation project addressing evidence–practice gap in the management of choledocholithiasis. The rationale is clinically strong, the multicenter national scope is a major strength, and the manuscript is clearly written overall. The protocol also appropriately frames itself as an implementation project using CFIR, MOST, and a before–after design across participating hospitals. Because this is a protocol, result-oriented StaRI items are understandably not yet applicable. The following are suggestions for both major and minor revisions:

First, thank you for your kind words on our protocol. Second, we value your elaborate comments on our manuscript and addressed all of them in the revised version. Hereunder, we responded to the suggestions made:

Major revisions

1. It would be great to clarify and harmonize the analytic population across study phases.

This is the most important issue. In phase 1, the manuscript states that all patients undergoing ERCP for choledocholithiasis during the baseline period will be analyzed, whereas in phase 3 the evaluable population appears more closely linked to patients eligible for the intended care pathway and the primary endpoint is defined as cholecystectomy within 72 hours after successful ERCP. As written, it is not fully clear whether the denominator is identical across phases, particularly with respect to failed ERCP, patients with contraindications to surgery, and transferred patients. This raises concern about comparability in the before–after analysis. The manuscript would be strengthened by defining one consistent analytic population and applying it identically in both phases.

Thank you for this important comment. We agree that comparability of the denominator across phase is essential for the validity of the before-after analysis. Therefore, we have harmonized the analytic population across both phases. For the primary analysis, the denominator will be applied identically in phase 1 and phase 3. This will consist of all eligible patients with a gallbladder in situ who underwent a first successful ERCP for choledocholithiasis. Patients with surgical contraindications to cholecystectomy will be registered but excluded from the primary outcome analysis in both phases. Patients who decline cholecystectomy or in whom cholecystectomy is deferred for non-surgical reasons

will remain included. Transferred patients will be assigned to the hospital where the successful ERCP was performed. Failed ERCP attempts will be described separately but will not be included in the primary denominator. We have adjusted the methods and statistical analysis section accordingly.

Revised version:

- *Methods, Targeted sites, population and eligibility criteria, lines 206-207, 215-217, 221-226*

2. Providing a more rigorous sample size justification and a more fully specified statistical analysis plan would be helpful for the potential readers and researchers.

The manuscript reports expected hospital participation and projected patient numbers and states that mixed-effects modeling will be used to account for clustering and case-mix. However, the sample size justification remains pragmatic rather than analytical, and the statistical section would benefit from more detail. In particular, the authors should specify the model structure more explicitly, including fixed and random effects, handling of hospital-level clustering, prespecified confounders, missing-data strategy, and planned sensitivity analyses. Since the study is non-randomized and multicenter, this level of detail is important for reader confidence.

We agree with the reviewer that the statistical analysis plan required more detail. We have therefore included a more detailed sample size justification and have adjusted and specified our statistical analysis plan.

A formal sample size calculation is less straightforward in this setting, because the current study is a national implementation project with a predefined adherence target. Rather than a conventional trial designed primarily to test a treatment effect. However, for illustrative reasons we have added a conventional superiority calculation. As the expected increase from approximately 10% to 85% adherence is large, only a small number of patients is required (n=7). To provide a more informative justification, we added a precision calculation for the adherence rate. This shows that the planned sample provides sufficient precision to estimate national adherence around the implementation target of 85%.

In addition, the mixed-effects logistic regression model has been further specified. This now includes period as fixed effect and hospital as random intercept. We also list the prespecified case-mix variables and describe the missing data strategy using multiple imputation. Also, we have added prespecified sensitivity analyses.

Revised version:

- *Methods, Sample Size, lines 228-234, 239-245*
- *Outcome measures and predefined statistical analysis plan, Primary objectives' statistical plan, lines 272-273, 276-291*

3. Please prespecify fidelity, adaptation, maintenance, and registration information more clearly.

The supplement notes that adoption, implementation, and maintenance will be evaluated, and the main text states that local guidelines will be reviewed every four weeks and adjusted if necessary. However, the protocol does not yet clearly define how fidelity to the implementation strategy will be measured, how adaptations will be documented, or which process metrics will indicate successful implementation. In addition, the StaRI checklist includes study registration under general reporting items, but a registration platform/number or a clear explanation for non-registration is not readily visible in the manuscript. These points should be addressed explicitly.

We have included a more detailed explanation of the implementation process metrics in the supplemental files. Success will be defined by RE-AIM indicators at the hospital level, including protocol development, use, and fidelity. Fidelity will be assessed by whether activities were delivered as intended. Adaptations will be documented throughout all phases via ongoing communication with hospitals (monthly updates, emails, webinars, and feedback), and adherence will be tracked through real-time feedback and protocol change records. Maintenance will be measured by surveys on protocol use and intention to maintain after project completion, and by including timely cholecystectomy as a quality criterion in relevant Dutch registries.

We added a study registration statement: this study was not prospectively registered in a clinical trial registry; however, the complete protocol is presented in this manuscript conform StaRI guidelines for transparency and reproducibility.

Revised version:

- *Supplementary Appendices, Supplement 1, Secondary objectives, lines 116, 124-135*
- *Main text, Ethics and Dissemination, General, lines 340-343*

Minor revisions

1. Adding an outcome definition table would improve readability.

Several outcomes are defined across the main manuscript and supplement, including successful ERCP, morbidity, surgical complications, cost outcomes, and implementation outcomes. A single consolidated table summarizing each outcome, its definition, timepoint, data source, and unit of analysis would improve readability and reduce ambiguity.

We added a supplementary table with all the outcomes mentioned in the manuscript.

Revised version

- *Supplementary appendices, Supplement 1, Table 1, line 59*

2. Please specify the contribution of patient and public involvement.

The manuscript states that the Dutch Liver Patient Association provided input on patient-related outcomes. It would be helpful to specify what, concretely, changed as a result of that input.

The Dutch Liver Patient Association was involved from study design onwards. They were asked to ensure patient perspective and contribute to the development of centralised patient information. They shared their thoughts during the project concept presentation and gave feedback on the trial protocol. Their input confirmed that the Dutch-TIMELINESS project is

relevant for patients. We cannot determine which outcomes were added solely by the Dutch Liver Patient Association.

Revised version

- *Methods, Patient and Public Involvement Statement, lines 248-252*

3. There are rooms for improvement regarding terminology and consistent use of words.

Examples include consistent use of “StaRI” capitalization, clearer wording around hospital numbers and ERCP capability, and minor language edits throughout. These are not major concerns, but they would improve readability of the protocol.

I look forward to implementation of the current protocol and thus future improvement in the management of choledocholithiasis.

We have proofread and edited the entire manuscript to further improve readability and clarity, especially regarding terminology and consistent use of words. The section about hospital numbers and ERCP capability has been adjusted.

VERSION 2 - REVIEW

Reviewer **1**

Name **Han, Jimin**

Affiliation **Daegu Catholic University School of Medicine, Department of Internal Medicine**

Date **08-Jun-2026**

COI

The revised manuscript has improved greatly in clarity and readability.

The authors' efforts are deeply appreciated.
